# Characterisation and sustainability evaluation of cereals and pulses formal seed production systems in Cameroon

**DOI:** 10.1371/journal.pone.0336670

**Published:** 2025-11-14

**Authors:** Moise Harnold Fotso Ngangoua, Eric Bertrand Kouam

**Affiliations:** Genetics, Biotechnology, Agriculture and Plant Production Research Unit, Department of Crop Sciences, Faculty of Agronomy and Agricultural Sciences, University of Dschang, Dschang, Cameroon; Makerere University College of Natural Sciences, UGANDA

## Abstract

Cereals and pulses are essential commodities for human nutrition in Africa. Seed quality is the vital input and precondition for good agricultural production. Good seeds are mainly supplied by the formal system, which is struggling to ensure its sustainability in Africa. Providing solutions to this systemic problem first requires making a diagnosis. This study aims to characterize the cereal and pulse formal seed production system in Cameroon. The study was conducted in the Sudano-Sahelian (SSZ) and Western highlands (WHZ) agroecological zones (AEZs) of Cameroon and consisted of a triangulation of information: (1) documentary research, (2) semi-structured interviews with seed certification managers and, (3) administration of questionnaires to seed producers. Cross-tabulations and chi-square tests revealed a statistically significant differential distribution of many seed system variables between AEZs, botanical category, cereal types, and legume types. Results reveal that seed production activity is strongly dominated by men with however a considerable proportion of women observed in WHZ (23.3%) compared to SSZ (1.5%). In this latter zone, the declared areas are much larger than in WHZ. Therefore, in order to preserve the purity of crops intended to be seeds, spatial isolation (72.2%) is mainly observed in SSZ against temporal isolation (58.1%) in WHZ. Diversity estimates of varieties and species in seed production were significantly higher in SSZ [H’ (variety) =2.86; H’ (species) = 1.68); richness (variety = 25); richness (species) = 6] compared to WHZ [H’ (variety) =2.31; H’ (species) = 1.13); richness (variety = 19); richness (species) = 6]. Seeds are mostly produced in monocropping system in both zones (95% in SSZ and 62% in WHZ) in comparison to intercropping system. Average yields are relatively low in both AEZs (1 to 2 t/ha for cereals and less than 32 1 t/ha for pulses). Although the low yield coupled with the unsold of all the seeds produced during the cropping season weakens the production system, the marked diversity observed in both AEZs is important for strengthening the sustainability of the seed production system in Cameroon.

## Introduction

The world population reached 8 billion in 2022 [1]. The Word Health Organization estimated in 2023, that 733 million people are facing hunger worldwide, meaning one over 11 people [2]. This figure reflects a stagnation in progress towards Sustainable Development Goal 2 that is zero hunger, with levels of undernutrition similar to those in 2008–2009 [2]. Africa is particularly affected by this crisis, with 20.4% of its population undernourished [3]. This is partly due to the fact that most households in Africa in their livelihoods depend from small-scale, mainly rain-fed agricultural systems, highly vulnerable to climate shocks [4]. The current crisis at the international level amplified by conflicts and climate change requires urgent strategies and coordinated efforts to ensure food security [3]. Cereals and pulses are essential for human and animal nutrition in Africa [5]. Cereals provide, approximately 70% of dietary energy and considerable protein as well as essential vitamins and minerals such as iron and zinc [6,7]. More specifically, maize and rice are essential staple foods worldwide and contributing significantly to human nutrition in Africa [8]. Indeed, maize is a primary energy source providing more than 30% of total caloric intake in sub-Saharan Africa while rice is the staple food for more than 50% of the world’s population [9,10]. Pulses, rich in protein, fibers, and micronutrients, complement cereals in human nutrition with synergistic interaction [11]. Pulses are used in improving soil fertility through biological nitrogen fixation and they provide essential amino acids for dietary health benefits [12,13]. In this regard, while peanuts on the one hand offer high protein content (up to 27%), healthy fats and bioactive compounds that promote heart health and combat malnutrition especially in children, beans and cowpea on the other hand are rich in protein, carbohydrates and fiber, contributing to the energy needed and to the digestive health [14,15]. Ensuring food security and sustainability through the production of these different commodities involves seed security which is a cardinal point of food availability and safety. Indeed, seed security guarantees the availability, accessibility and quality of seeds necessary for agricultural production [16]. Seed of good quality is considered as the vital input and the prerequisite for good agricultural production. The production and distribution of seeds is ensured by two main systems namely the formal and the informal seed production system [17]. Formal seed systems account for around 20% of planted seed in Africa [17]. This system is regulated to ensure that seed retains its varietal identity and purity. The regulation processes involve research institutions and seed certification organisations. In Cameroon, seed production in the formal system is supervised by the Directorate for Regulation and Quality Control of Agricultural Inputs and Products. Seed certification activity carried out by this department can be broken down as follows: technical investigation, certification of seed production activity, crop declaration, seed royalty, inspection, quantity estimation, sampling, seed lot quality certification, storage, packaging, seed transaction register and marketing control. Formal seed system contributes significantly to food security by ensuring genetic purity and optimal seed quality. Conversely, the informal seed system, mainly used by smallholder farmers, allows for greater accessibility and adaptability, provide 80% of the seed supply in tropical Africa [17]. It promotes biodiversity and local resilience as farmers exchange seeds adapted to their needs [18,19]. Both systems are often integrated, which contributes to sustainability. They are essential for agricultural productivity and food security, particularly in developing countries [20,21]. Although the formal system ensures a more adequate supply of high-quality seeds, it faces several difficulties. Particularly in Africa, the formal seed production system is struggling to ensure its sustainability due to climate change and poor cultivation practices, which jeopardizes seed security [22,23]. The objective of this study is therefore to characterize and evaluate the sustainability of the formal cereal and pulse seed production system in the Sudano-Sahelian (SSZ) and Western highlands (WHZ) agroecological zones (AEZs) of Cameroon.

## Materials and methods

### Ethical considerations

The ethical approval for this research was granted by the Cameroonian Ethical Comity for Research with approval number No 93/26/02/2025/CE/CRERSH-OU/VP. Before taking part in the survey, a verbal consent agreement was obtained from each voluntary interviewee by the research team. These interviewees were therefore provided with detailed information about the study and face-to-face interviews were then conducted in order to facilitate effective understanding. Explanations and clarifications when required were provided by the research team comprising students and two personnels from the Ministry of Agriculture and Rural Development.

### Study zones

**Fig 1:**
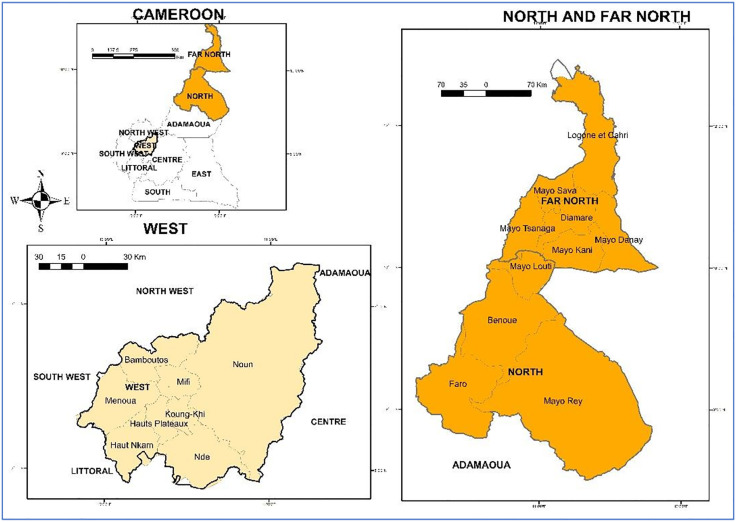
Map showing the survey localities of seed production systems in two AEZs of Cameroon. The map was generated using ArcGIS 10.3 software.

The study was conducted between 2022 and 2023 in two agroecological zones, the Soudano-Sahelian and Western Highlands sites of Cameroon. These two zones were chosen because they constitute the major seed production basins of the country. The Sudano-Sahelian zone is located between 8°36’ and 12°54’ North latitude, and between 12°30’ and 15°44’ East longitude, and covers an area of nearly 100,353 Km^2^. The climate there is characterized by monomodal rainfall extending from September to November. Rainfall is between 400 and 1200 mm per year and average temperatures vary between 28°C and 45°C. The Western Highlands area is located between 4°54’ and 6°36’ North latitude and between 9°18’ and 11°24’ East longitude and covers an area of approximately 31,192 km2. The climate is characterized by two seasons of unequal length. The rainy season lasts from March to November and the dry season from December to February. Average temperatures are lower (19°C), and rainfall is more abundant (1500–2000 mm per year). The map showing these two sites is shown in Fig 1.

### Methodological approach

The methodological approach consisted of a triangulation of information. First, a documentary search enabled to build up an information base necessary to address the subject and reach the resource persons. Secondly, semi-structured individual interviews were conducted with seed certification managers in the different agroecological study zones. The objective was to obtain information on the processes leading to the certification of a seed, and also to be introduced to seed producers. Finally, in a third step, questionnaire administrations were carried out among seed producers. The objective was to collect information on seed producers and companies, production and conservation practices, and assess the sustainability of the seed production system. The survey form consisted of open, closed and demographic questions. The questionnaire was pre-tested on six seed producers and revised accordingly; it included 57 questions subdivided into three sections namely: demographic information, production process and seed conservation procedure. The population size of the seed producers obtained from the seed certification services in the two agro-ecological zones was 392 individuals. The adjusted formula used to calculate the sample size for small populations was used: $n=\frac{n_{\text{0}}}{\text{1}+(\frac{n_{\text{0}}-\text{1}}{N})}$ where *n*_*0 *_*= 385* is the sample size recommended by Cochran [24]. With a population size of N = 392, the new adjusted sample size is therefore n = 194. But due to the difficulty in reaching some seed producers, only 176 individuals were interviewed. However, this sample size is much higher than the proportion of 20% that is commonly recommended [24]. In order to allow a better representation of the different subgroups in the sample, stratified sampling with proportional distribution was used [25].

### Statistical analysis

The information collected from the survey questionnaires was entered into the Excel 2013 spreadsheet and coded numerically. These data were then exported and analysed using IMB SPSS computer statistical program, version 23.0. Descriptive analyses were performed to express the frequency of observations as a percentage. The chi-square test at the 5% threshold was performed to assess the relationship between variables. In addition, a correlation following the multiple correspondence analysis (MCA) model was performed between variables. The richness and Shannon-Weaver diversity index (H) were calculated at the cultivar and at the species level. H for each cultivar and species was calculated as follow: $H=-\sum_{i=\text{1}}^{n}x_{i}{(x}_{i}$ with *x*_*i*_ representing the relative frequency of the *i*^*th*^ cultivar or species and *n* the total number of cultivars or species [26]. Comparisons of Shannon-Weaver diversity index between agro-ecological zones were performed using the Hutcheson t-test [27]. Multiple correspondence analysis was used to highlight the existing relationships between variables through coefficient of correlation. Positive correlation reflects similar characteristics while negative values explain contrasting characteristics between variables [28].

## Results

### Socio-demographic characteristics of seed producers

The results of the Socio-demographics characteristics of seed producers are presented in Table 1. The Table shows that the distribution of the gender carrying out the seed multiplication activity is a function of the agroecological zone (ꭕ^2^ = 24.2***). The activity is dominated by men with a proportion of 98.5% against 1.5% of women in the Sudano-Sahelian zone and 76.7% of men against 23.3% of women in the highlands zone (Table 1). The level of education of seed producers is independent on the AEZs (ꭕ^2^ = 1.9^NS^). The results show however that the majority of seed producers have a higher level of education with a proportion of 62.4% and 72.1% in the Sudano-Sahelian and western highlands zones respectively. Concerning the main occupation of the farmers, the chi-square test reveals a dependence with the AEZs (ꭕ^2^ = 88.3***). It is observed that in the Sudano-Sahelian zone the majority of farmers (57.1%) have seed production as their main occupation, without having received formal training in the agricultural field. In the highlands zone in contrary, although the majority of farmers (34.9%) have seed production as their main occupation, they have previously obtained training as agricultural technicians. As for the category of enterprise, it should be noted that it depends on the agro-ecological zone (ꭕ^2^ = 29.4**). Crop Research Centres (CRC), Seed Production Cooperative Societies (SPCS) and Common Initiative Groups (CIG) are the main business categories in the Sudano-Sahelian zone (35.0, 27.1 and 25.6% respectively) meanwhile, CIG (67.4%) is the main business category in the highlands zone. The declared farm area is dependent on the AEZs (ꭕ^2^ = 19.6***). Almost all producers in the highlands zone have a declared farm area of less than 2 ha (90.7%) with no producer having an area beyond 5 ha. In the Sudano-Sahelian zone in contrary, the declared farm area of less than 1 ha is the most representative (36.1%) although larger areas are also significantly represented. Higher declared farm areas, beyond 5 ha (14.3%) and even more than 10 ha (4.5%) are observed in this zone. As for the characteristics of seed producers and companies, the chi-square test at the 5% threshold reveals that a dependency relationship between the botanical category of seed produced and gender (ꭕ^2^ = 5.5**), main occupation of seed producer (ꭕ^2^ = 15.6*) and the seeds class produced (ꭕ^2^ = 6.6*). Several other socio-demographic parameters of seed producers do not show any dependency with the botanical category of seed, the type of cereal and the type of pulses produced. A higher proportion of women are found to be involved in the production of cereals seeds (10.5) compared to women who produce pulses seeds (1.4%).

**Table 1:** Characteristics of seed producers and companies in the SSZ and WHZ of Cameroon, expressed as percentage.

Variables	Modalities	Agro-ecological zone			Botanical category			Cereal				Pulse				
		SSZ	WHZ		Cereal	Pulse		Maize	Rice	Sorghum		Peanut	Soybean	Cowpea	Common bean	
		N = 133	N = 43		N = 105	N = 71		N = 60	N = 14	N = 31		N = 13	N = 14	N = 32	N = 12	
				Chi-square			Chi-square				Chi-square					Chi-square
Gender	Male	98.50	76.74	24.2^***^	89.52	98.59	5.5**	86.67	78.57	100.00	5.9*	100.00	100.00	100.00	91.67	5.0^NS^
	Female	1.50	23.26		10.48	1.41		13.33	21.43	0.00		0.00	0.00	0.00	8.33	
Level of study	Primary	17.29	9.30	1.9^NS^	17.14	12.68	2.6^NS^	13.33	35.71	16.13	8.3^NS^	23.08	14.29	12.50	0.00	4.8^NS^
	Secondary	20.30	18.60		22.86	15.49		23.33	0.00	32.26		23.08	14.29	15.63	8.33	
	University	62.41	72.09		60.00	71.83		63.33	64.29	51.61		53.85	71.43	71.88	91.67	
Farm manager qualification	Agricultural scientist	35.34	27.91	88.3^***^	25.71	45.07	15.6^*^	20.00	50.00	25.81	23.3^NS^	38.46	42.86	40.63	66.67	30.8^NS^
	Agricultural technician	1.50	34.88		14.29	2.82		21.67	14.29	0.00		0.00	0.00	0.00	16.67	
	Seed farmer	57.14	4.65		43.81	45.07		36.67	21.43	67.74		53.85	57.14	53.13	0.00	
	Farm manager	2.26	0.00		1.90	1.41		1.67	0.00	3.23		0.00	0.00	3.13	0.00	
	Farmer	3.76	16.28		9.52	2.82		11.67	14.29	3.23		7.69	0.00	3.13	0.00	
	Agricultural engineer	0.00	6.98		2.86	0.00		5.00	0.00	0.00		0.00	0.00	0.00	0.00	
	Zootechnician	0.00	4.65		0.95	1.41		1.67	0.00	0.00		0.00	0.00	0.00	8.33	
	Nurse	0.00	4.65		0.95	1.41		1.67	0.00	0.00		0.00	0.00	0.00	8.33	
Business Category	Research center	35.34	27.91	29.4^***^	25.71	45.07	8.4^NS^	20.00	50.00	25.81	16.3^NS^	38.46	42.86	40.63	66.67	9.9^NS^
	Individual	9.77	0.00		6.67	8.45		5.00	14.29	6.45		15.38	7.14	9.38	0.00	
	SPCS	27.07	4.65		25.71	15.49		20.00	21.43	38.71		23.08	14.29	18.75	0.00	
	Farm	2.26	0.00		1.90	1.41		1.67	0.00	3.23		0.00	0.00	3.13	0.00	
	CIGs	25.56	67.44		40.00	29.58		53.33	14.29	25.81		23.08	35.71	28.13	33.33	
Seed Class	Certified	62.41	72.09	1.3^NS^	72.38	53.52	6.6^*^	78.33	50.00	70.97	4.6^NS^	61.54	57.14	56.25	33.33	2.5^NS^
	Foundation	37.59	27.91		27.62	46.48		21.67	50.00	29.03		38.46	42.86	43.75	66.67	
Declared farm area (ha)	≤1	36.09	67.44	19.6^***^	40.95	47.89	7.7^NS^	45.00	35.71	35.48	11.1^NS^	53.85	71.43	28.13	66.67	15.9^NS^
	]1;2]	19.55	23.26		16.19	26.76		18.33	7.14	16.13		23.08	14.29	31.25	33.33	
	]2;5]	25.56	9.30		23.81	18.31		21.67	50.00	16.13		23.08	14.29	25.00	0.00	
	]5;10]	14.29	0.00		14.29	5.63		11.67	7.14	22.58		0.00	0.00	12.50	0.00	
	>10	4.51	0.00		4.76	1.41		3.33	0.00	9.68		0.00	0.00	3.13	0.00	

***: Significant at 0.001 probability level, **: Significant at 0.01 probability level, *: Significant at 0.05 probability level, ^NS^: Not significant.

### Seed production practices

Data on seed production practice are presented in Table 2. The majority of farmers in both agroecological zones revealed that original seeds come from the stations of the Institute of Agricultural Research for Development (IRAD). However, the botanical category of seed produced depends on the origin (ꭕ^2^ = 7.9**). A considerable proportion of cereal seeds come from farms (16.2%) compared to pulses (2.8%). Regarding the useful surface area, its distribution depends on the AEZs (ꭕ^2^ = 20.7***), the botanical category of seed (ꭕ^2^ = 74.6***), the type of cereals (ꭕ^2^ = 98.1***) and even the pulses produced (ꭕ^2^ = 46.9***). Large areas of between 0.3 and 0.4 m^2^ per pocket are mainly used in the Sudano-Sahelian zone (50.4%), compared to areas of 0 to 0.1 m^2^ per pocket in the Highlands zone (37.2%). With the exception of rice, cereals tend to occupy larger areas, between 0.3 and 0.4 m^2^per pocket (66.7%), compared to pulses, which tend to use only 0 to 0.1 m^2^ per pocket (59.2%). However, rice, which is a cereal, mainly uses these small useful areas (85.7%). The distribution of seed density depends on the AEZs (ꭕ^2^ = 62.3***). High seed densities (20–25 seeds/m2) are reported for the Sudano-Sahelian zone (29.3%), whereas lower seed densities (1–5 seeds/m2) which are mainly observed in the highlands area (44.2%). The practice of thinning depends on the AEZs (ꭕ^2^ = 45.3***), with this practice not observed in the highlands area. The distribution of planting density is independent on the AEZs (ꭕ^2^ = 5.6^NS^). The planting density between 1 and 50,000 plants/ ha is the most representative in both AEZs. However, the distribution of planting density depends on the botanical category of seed (ꭕ^2^ = 98.0***), the type of cereals (ꭕ^2^ = 111.6***) and the type of pulses produced (ꭕ^2^ = 52.5***). Therefore, the proportion of the most represented cereal planting density is between 1 and 50,000 plants/ ha (65.7%) compared to more than 250,000 plants/ ha for pulses (29.6%). The distribution of the type of isolation depends on the AEZs (ꭕ^2^ = 91.6***), the botanical category of seed (ꭕ^2^ = 24.1***), and the pulses produced (ꭕ^2^ = 48.3***). It is independent of the type of cereals produced (ꭕ^2^ = 10.9^NS^). The most represented type of isolation in the Sudano-Sahelian zone is spatial isolation (72.2%) and temporal isolation (58.1%) in the highlands zone. Temporal isolation is non-existent in the Sudano-Sahelian zone. We observe rather in this zone a specificity, the practice of seed sharing with close neighbours, i.e., offer to the neighbours the sharing of seeds (5.3%). We observe that the spatial isolation represents the highest proportions for cereals (74.3%) or pulses (47.9%). However, isolation by sharing seeds with neighbours is not observed in pulses, but cultural isolation (practice of sowing different species in the seed field from the one cultivated by neighbours) in the latter has a considerable representativeness (31.0%). Common beans in particular benefit much more from temporal isolation with 91.7% of respondents who present this practice as a choice of isolation. Concerning the timing of campaign entry and its duration, their distribution depends on the agroecological zone, ꭕ^2^ = 35.1*** and ꭕ^2^ = 15.3*** respectively. Therefore, in both AEZs, sowing is mainly carried out at the beginning of the season with proportions of 84.2% and 46.5% in the Sudano-Sahelian and highlands zones respectively. However, in the highlands zone, a considerable proportion of sowing after the start of the rains is observed (41.9%) against a relatively low proportion in the Sudano-Sahelian zone (11.3%). This is due to the relatively short duration of the rainy season in the Sudano-Sahelian zone compared to the highlands zone. This fact has an impact on the duration of the seed production season which translates into an average duration of 5 months (47.4%) in the Sudano-Sahelian zone against 4 months in the highlands zone (62.8%). But the distribution of the duration of the season does not depend on the botanical category of seed (ꭕ^2^ = 2.0^NS^). The duration of the most representative campaign is 4 months for cereals (44.8%) and for pulses (40.8%). The distribution of the timing of campaign entry depends on the botanical category of seed (ꭕ^2^ = 34.9***). The majority of producers enter to campaign at the beginning of the rainy season, whether for cereals (90.5%) or pulses (52.1%). However, a considerable proportion of pulse producers enter to campaign after the start of the rains (38.0%).

**Table 2:** Characteristics of seed production practices in the SSZ and WHZ of Cameroon, expressed as percentage.

Variables	Modalities	Agro-ecological zone			Botanical category			Cereal				Pulse				
		SSZ	WHZ		Cereal	Pulse		Maize	Rice	Sorghum		Peanut	Soybean	Cowpea	Common bean	
		N = 133	N = 43		N = 105	N = 71		N = 60	N = 14	N = 31		N = 13	N = 14	N = 32	N = 12	
				Chi-square			Chi-square				Chi-square					Chi-square
Origin of mother seeds	IRAD	90.23	86.05	0.6^NS^	83.81	97.18	7.9^**^	83.33	100.00	77.42	3.6^NS^	92.31	100.00	96.88	100.00	1.9^NS^
	Farm	9.77	13.95		16.19	2.82		16.67	0.00	22.58		7.69	0.00	3.13	0.00	
Plant occupation (pocket/m^2^)	≤0.1	28.57	37.21	20.7^***^	11.43	59.15	74.6^***^	0.00	85.71	0.00	98.1^***^	100.00	85.71	15.63	100.00	46.9^***^
	]0.1;0.2]	16.54	20.93		10.48	28.17		13.33	14.29	3.23		0.00	14.29	56.25	0.00	
	]0.2;0.3]	0.00	11.63		4.76	0.00		8.33	0.00	0.00		0.00	0.00	0.00	0.00	
	]0.3;0.4]	50.38	27.91		66.67	12.68		73.33	0.00	83.87		0.00	0.00	28.13	0.00	
	]0.4;0.5]	4.51	2.33		6.67	0.00		5.00	0.00	12.90		0.00	0.00	0.00	0.00	
Sowing density (grains/m^2^)	≤5	1.50	44.19	62.3^***^	18.10	2.82	41.1^***^	31.67	0.00	0.00	145.1^***^	0.00	0.00	6.25	0.00	55.5^***^
	]5;10]	27.82	11.63		34.29	8.45		55.00	0.00	9.68		0.00	0.00	18.75	0.00	
	]10;15]	18.05	6.98		5.71	29.58		10.00	0.00	0.00		61.54	0.00	40.63	0.00	
	]15;20]	1.50	0.00		0.00	2.82		0.00	0.00	0.00		15.38	0.00	0.00	0.00	
	]20;25]	29.32	9.30		22.86	26.76		1.67	0.00	74.19		0.00	35.71	31.25	33.33	
	>25	21.80	27.91		19.05	29.58		1.67	100.00	16.13		23.08	64.29	3.13	66.67	
Thinning	Yes	58.65	0.00	45.3^***^	63.81	15.49	40.1^***^	56.67	21.43	96.77	26.8^***^	0.00	14.29	28.13	0.00	8.5^*^
	No	41.35	100.00		36.19	84.51		43.33	78.57	3.23		100.00	85.71	71.88	100.00	
Planting density (plants/ha)	≤50000	39.85	44.19	5.6^NS^	65.71	4.23	98.0^***^	81.67	0.00	64.52	111.6^**^	0.00	0.00	9.38	0.00	52.5^***^
	]50000;100000]	17.29	11.63		19.05	11.27		15.00	0.00	35.48		0.00	0.00	25.00	0.00	
	]100000;150000]	12.78	6.98		1.90	25.35		3.33	0.00	0.00		61.54	0.00	31.25	0.00	
	]150000;200000]	4.51	0.00		0.00	8.45		0.00	0.00	0.00		15.38	14.29	6.25	0.00	
	]200000;250000]	8.27	9.30		0.00	21.13		0.00	0.00	0.00		0.00	21.43	25.00	33.33	
	>250000	17.29	27.91		13.33	29.58		0.00	100.00	0.00		23.08	64.29	3.13	66.67	
Type of isolation	Spatial	72.18	37.21	91.6^***^	74.29	47.89	24.1^***^	68.33	92.86	77.42	10.9^NS^	30.77	64.29	65.63	0.00	48.3^***^
	Seed sharing	5.26	0.00		6.67	0.00		5.00	7.14	9.68		0.00	0.00	0.00	0.00	
	Cultural	22.56	4.65		9.52	30.99		10.00	0.00	12.90		53.85	28.57	31.25	8.33	
	Temporal	0.00	58.14		9.52	21.13		16.67	0.00	0.00		15.38	7.14	3.13	91.67	
Timing of production campaign	Early	0.00	9.30	35.1^***^	1.90	2.82	34.9^***^	3.33	0.00	0.00	8.9^NS^	0.00	0.00	0.00	16.67	26.2^**^
	Regular	84.21	46.51		90.48	52.11		88.33	92.86	93.55		69.23	64.29	59.38	0.00	
	Semi-late	11.28	41.86		5.71	38.03		8.33	7.14	0.00		30.77	35.71	28.13	75.00	
	Late	4.51	2.33		1.90	7.04		0.00	0.00	6.45		0.00	0.00	12.50	8.33	
Campaign duration	3 months	15.79	23.26	15.3^***^	14.29	22.54	2.0^NS^	18.33	7.14	9.68	11.1^*^	15.38	21.43	31.25	8.33	22.5^**^
	4 months	36.84	62.79		44.76	40.85		43.33	78.57	32.26		53.85	42.86	15.63	91.67	
	5 months	47.37	13.95		40.95	36.62		38.33	14.29	58.06		30.77	35.71	53.13	0.00	

***: Significant at 0.001 probability level, **: Significant at 0.01 probability level, *: Significant at 0.05 probability level, ^NS^: Not significant.

### Seed post-production conducts

Data on seed post production are presented in Table 3 which shows that many activities are implemented to ensure seed quality. Chi-square test reveals a dependence between the distribution of the modalities of the drying technology parameters and drying time with AEZs (ꭕ^2^ = 66.4*** and ꭕ^2^ = 77.6***) and the botanical category of seed (ꭕ^2^ = 29.5*** and ꭕ^2^ = 18.3***). Although drying on the field has the most marked proportions in the Sudano-Sahelian zone (65.4%) and highlands (39.5%). It does observe the existence of the use of cribs dryer (23.3%) and granaries (14.0%) as drying technology in the highlands zone. These are non-existent in the Sudano-Sahelian zone. This on-field drying is strongly represented with cereals (64.8%) and pulses (50.7%). However, the use of open-air drying in pulses is highly representative (33.8%) compared to cereals (6.7%). The distribution of drying time depends on the AEZs, it is observed that the most representative range of drying time in the Sudano-Sahelian zone is 2–5 weeks (63.2%). In the highlands zone, on the other hand, the range is shared between 2–5 weeks of drying (41.9%) and beyond 8 weeks (41.9%). Also, the most marked drying time margin in cereals (59.0%) and pulses (56.3%) is 2–5 weeks. However, a considerable proportion of pulses (40.8%) have a short drying time margin of between 1 and 2 weeks. The modalities of the technology used for seed calibration, showed dependence with AEZs (ꭕ^2^ = 29.8***), the botanical category of seed (ꭕ^2^ = 33.1***), types of cereals (ꭕ^2^ = 62.2***) and pulses produced (ꭕ^2^ = 39.4***). Using a sieve is the tool mainly used for calibration in the Sudano-Sahelian zone (67.7%) and in the highlands zone (58.1%). In this latter zone, winnowing has a marked representativeness (34.9%) compared to the Sudano-Sahelian zone (5.3%). The Sahel uses technologies such as the cleaner-separator and manual sorting which are non-existent in the highlands zone. However, in both AEZs, there are seed multipliers who do not calibrate their seeds. Not calibrating seeds is more pronounced in the Sudano-Sahelian zone (17.3%) than in the highlands zone (7.0%), and only appears in cereal production (24.8%). The sieve is mainly used for both cereal (60.0%) and pulse (73.2%) cultivation. While the sieve is mainly used for calibrating maize (83.3%) and rice (50.0%), sorghum on the other hand is generally not calibrated (64.5%). In pulses, the proportion of sieve use is higher for peanuts (69.2%), soybeans (85.7%) and cowpeas (90.6%) while winnowing is mainly used for beans (83.3%). In terms of products used for seed treatment against pests, statistical analysis shows a dependence with AEZs (ꭕ^2^ = 132.9***), botanical category of seed (ꭕ^2^ = 42.2***), type of cereals (ꭕ^2^ = 37.6***) and pulses produced (ꭕ^2^ = 50.3***). In the Sudano-Sahelian zone, the majority of producers do not use any synthetic product for seed treatment (85.7%) and pest control (39.1%). In the highlands zone in contrary, a considerable proportion of producers use the insecticide “Antouka” (Pirimiphosphomethyl + permethrine) at 41.9% for seed treatment, and”cats” at 41.9% for rodent control. Concerning seed botanical category, cereals and pulses have a high proportion of non-submission to treatment, 61.9% and 71.8% respectively. However, “Momtaz” (Imidaclopride + Thirame) which is an insecticide-fungicide is used for the treatment of cereals (23.8%), while the insecticide “antouka” is significantly used for pulses (18.3%). More specifically, while “Momtaz” is widely used for the treatment of corn (40%), “antouka” is used for peanuts (15.4%), soybeans (7.1%) and common beans (75.0%). But “Aluminum phosphide” which is a fumigant pesticide is remarkably used in the treatment of cowpea (15.6%). Concerning the seed conservation techniques used, two common techniques are observed, namely ambient and hermetic conservation. The distribution of the modalities of this parameter depends on the agroecological zone (ꭕ^2^ = 12.2***), the botanical category of seed (ꭕ^2^ = 56.3***) and the pulses produced (ꭕ^2^ = 67.1***). In the highlands zone, 100% of respondents use ambient storage compared to 76.7% in the Sudano-Sahelian zone. Cereals are fully stored ambiently, while pulses are divided between ambient (56.3%) and hermetic (43.7%) storage. Hermetic storage using “PICS” bags is only observed for cowpea (96.9%) and in the Sudano-Sahelian zone (23.3%). The availability of a storage warehouse depends on the agroecological zone (ꭕ^2^ = 41.2***), the botanical category of seed (ꭕ^2^ = 7.9**), type of cereals (ꭕ^2^ = 8.9*) and pulses produced (ꭕ^2^ = 10.1**). As a result, a higher proportion of producers in the Sudano-Sahelian zone (97.7%) have a storage warehouse compared to producers in the highlands zone (62.8%). The proportion of stored pulses (97.2%) is higher than that of cereals (83.8%). The distribution of the storage duration categories depends on the AEZs (ꭕ^2^ = 67.4***), the botanical category of seed (ꭕ^2^ = 11.0*), cereals (ꭕ^2^ = 29.9**) and pulses (ꭕ^2^ = 27.0*). The average storage duration is 5 months in both the Sudano-Sahelian zone (57.1%) and Western highlands (48.8%). Considerable proportions of long storage periods of almost 7 months are observed in the Sudano-Sahelian zone (24.1%) while considerable shorter storage periods of 3 months in the highlands zone (25.6%).

**Table 3:** Characteristics of seed post-production variables in the SSZ and WHZ of Cameroon, expressed as a percentage.

Variables	Modalities	Agro-ecological zone			Botanical category			Cereal				Pulse				
		SSZ	WHZ		Cereal	Pulse		Maize	Rice	Sorghum		Peanut	Soybean	Cowpea	Common bean	
		N = 133	N = 43		N = 105	N = 71		N = 60	N = 14	N = 31		N = 13	N = 14	N = 32	N = 12	
				Chi-square			Chi-square				Chi-square					Chi-square
Drying technology	On-field drying	65.41	39.53	66.4^***^	64.76	50.70	29.5^***^	50.00	85.71	83.87	19.6^NS^	23.08	35.71	53.13	91.67	29.1^**^
	Open-air drying	19.55	11.63		6.67	33.80		8.33	0.00	6.45		76.92	35.71	28.13	0.00	
	On field and open-air drying	15.04	4.65		11.43	14.08		11.67	14.29	9.68		0.00	28.57	18.75	0.00	
	On fields and granary	0.00	2.33		0.95	0.00		1.67	0.00	0.00		0.00	0.00	0.00	0.00	
	Granary	0.00	13.95		4.76	1.41		8.33	0.00	0.00		0.00	0.00	0.00	8.33	
	Crib	0.00	23.26		9.52	0.00		16.67	0.00	0.00		0.00	0.00	0.00	0.00	
	On fields and crib	0.00	4.65		1.90	0.00		3.33	0.00	0.00		0.00	0.00	0.00	0.00	
Drying duration (week)	≤ 2	35.34	4.65	77.6^***^	19.05	40.85	18.3^***^	1.67	78.57	25.81	58.1^***^	53.85	35.71	53.13	0.00	16.8^NS^
	]2;5]	63.16	41.86		59.05	56.34		60.00	21.43	74.19		46.15	64.29	43.75	91.67	
	]5;8]	1.50	11.63		5.71	1.41		10.00	0.00	0.00		0.00	0.00	3.13	0.00	
	> 8	0.00	41.86		16.19	1.41		28.33	0.00	0.00		0.00	0.00	0.00	8.33	
Calibration technology	Sieve	67.67	58.14	29.8^***^	60.00	73.24	33.1^***^	83.33	50.00	19.35	62.2^***^	69.23	85.71	90.63	16.67	39.4^***^
	Winnowing	5.26	34.88		7.62	19.72		0.00	21.43	16.13		15.38	7.14	3.13	83.33	
	Cleaner-separator	5.26	0.00		6.67	0.00		11.67	0.00	0.00		0.00	0.00	0.00	0.00	
	Manual sorting	4.51	0.00		0.95	7.04		1.67	0.00	0.00		15.38	7.14	6.25	0.00	
	None	17.29	6.98		24.76	0.00		3.33	28.57	64.52		0.00	0.00	0.00	0.00	
Seed treatment chemicals	None	85.71	4.65	132.9^**^	61.90	71.83	42.2^***^	38.33	92.86	93.55	37.6^***^	84.62	92.86	81.25	8.33	50.3^***^
	Aluminium phosphide	3.76	0.00		0.00	7.04		0.00	0.00	0.00		0.00	0.00	15.63	0.00	
	Momtaz	7.52	34.88		23.81	0.00		40.00	7.14	0.00		0.00	0.00	0.00	0.00	
	Malagrain	3.01	0.00		3.81	0.00		3.33	0.00	6.45		0.00	0.00	0.00	0.00	
	Momtaz and poudrox	0.00	13.95		5.71	0.00		10.00	0.00	0.00		0.00	0.00	0.00	0.00	
	Poudrox	0.00	4.65		0.00	2.82		0.00	0.00	0.00		0.00	0.00	0.00	16.67	
	Antouka	0.00	41.86		4.76	18.31		8.33	0.00	0.00		15.38	7.14	3.13	75.00	
Conservation technique	Ambient	76.69	100.00	12.2^***^	100.00	56.34	55.6^***^	100.00	100.00	100.00		100.00	100.00	3.13	100.00	67.1^***^
	Hermetic	23.31	0.00		0.00	43.66		0.00	0.00	0.00		0.00	0.00	96.88	0.00	
Mean seed conservation time	2 months	0.00	16.28	67.4^***^	4.76	2.82	11.0^*^	8.33	0.00	0.00	29.9^**^	0.00	0.00	0.00	16.67	27.0^*^
	3 months	0.00	25.58		9.52	1.41		16.67	0.00	0.00		0.00	0.00	0.00	8.33	
	4 months	3.01	2.33		0.95	5.63		0.00	7.14	0.00		0.00	0.00	12.50	0.00	
	5 months	57.14	48.84		49.52	63.38		48.33	21.43	64.52		61.54	64.29	62.50	66.67	
	6 months	15.79	4.65		13.33	12.68		8.33	21.43	19.35		15.38	7.14	18.75	0.00	
	7 months	24.06	2.33		21.90	14.08		18.33	50.00	16.13		23.08	28.57	6.25	8.33	
Warehouse availability	Yes	97.74	62.79	41.2^***^	83.81	97.18	7.9^**^	78.33	71.43	100.00	8.9^*^	100.00	100.00	100.00	83.33	10.1^**^
	No	2.26	37.21		16.19	2.82		21.67	28.57	0.00		0.00	0.00	0.00	16.67	
Rodent control means	None	39.10	9.30	94.5^***^	33.33	29.58	18.3^*^	31.67	50.00	29.03	17.1^NS^	23.08	42.86	37.50	0.00	55.1^***^
	Rat poison and trap	21.05	0.00		16.19	15.49		11.67	21.43	22.58		15.38	7.14	25.00	0.00	
	Rat poison and fumigant	7.52	0.00		7.62	2.82		5.00	0.00	16.13		0.00	0.00	6.25	0.00	
	Rat poison	25.56	23.26		25.71	23.94		30.00	7.14	25.81		46.15	28.57	21.88	0.00	
	Rat poison and cat	5.26	6.98		4.76	7.04		5.00	0.00	6.45		0.00	14.29	6.25	8.33	
	Cat	0.00	41.86		3.81	19.72		5.00	7.14	0.00		15.38	7.14	3.13	83.33	
	Rat poison. cat and trap	1.50	13.95		7.62	0.00		10.00	14.29	0.00		0.00	0.00	0.00	0.00	
	Trap and cat	0.00	4.65		0.95	1.41		1.67	0.00	0.00		0.00	0.00	0.00	8.33	

***: Significant at 0.001 probability level, **: Significant at 0.01 probability level, *: Significant at 0.05 probability level, ^NS^: Not significant.

### Sustainability of the seed production system

Data on the sustainability of the seed production system is presented in Table 4. The distribution of distances between the seed production and storage site is independent of the AEZs. However, the distance margin between 1 and 5 km is the most representative in the Sudano-Sahelian zone (51.9%) and in the highlands zone (44.2%). This same distance margin is the most representative in cereals production (41.0%) and pulses (63.4%); with however a distribution of the different modalities depending on the botanical category (ꭕ^2^ = 9.5*, Table 5). The distribution of the quantity of seed used per hectare depends on the AEZs (ꭕ^2^ = 49.3***), the botanical category (ꭕ^2^ = 39.8***), the cereals (ꭕ^2^ = 105.7***) and the pulses produced (ꭕ^2^ = 59.5***). Thus, seed quantities between 10 and 20 kg per hectare are regularly used in the Sudano-Sahelian zone (65.4%) and in the highlands zone (44.2%). However, in the latter zone, there is a high proportion of producers who use seed quantities greater than 50 kg per hectare (41.9%). The use of these large quantities of seeds is more marked in the production of pulses seeds (26.8%) compared to cereals (3.8%). The yield depends on AEZs (ꭕ^2^ = 39.3***), botanical category of seeds (ꭕ^2^ = 53.9***), the cereals cultivated (ꭕ^2^ = 34.0***) and the pulses produced (ꭕ^2^ = 29.5**). As a result, yields between 1 and 2 t/ha are mainly observed in the Sudano-Sahelian zone (54.9%) and in the highlands zone (41.9%). However, while significant proportions (23%) of yields below 1 t/ha are observed in the Sudano-Sahelian zone, larger proportions of yields above 2 t/ha are observed in the highlands zone. Cereals have an average yield of between 1 and 2 t/ha, while pulses have a greater proportion of yields below 1 t/ha (45.1%). However, beans in particular have a greater proportion of yields between 1 and 2 t/ha (41.7%). There is no relationship between the selling price of seeds and the AEZs (ꭕ^2^ = 2.0^NS^). However, there is a greater proportion of the price of a kg of seeds ranging between 900 and 1200 CFA francs in the Sudano-Sahelian zone (39.1%) and in the highlands zone (41.9%). The selling price of seeds is dependent on the botanical category of seed (ꭕ^2^ = 107.9***), cereals seeds (ꭕ^2^ = 90.4***) and pulses grains produced (ꭕ^2^ = 17.5*). Cereals seeds are less expensive compared to pulses seeds. Rice seeds among cereals are the most expensive and between pulses, peanuts seeds are highly expensive with the price per kg of seeds exceeding 1200 FCFA. The majority of seed producers in the highlands zone (88.4%) sell all of their seed. Conversely, in the Sudano-Sahelian zone, the majority of multipliers (59.4%) indicate that they do not sell all of their produced seed. Rice and groundnuts have the highest proportions of seeds that are not fully marketed, 71.0% and 61.5% respectively. Unmarketed seeds are generally redirected towards human consumption. The number of inspections during the seed production period depends on the AEZs (ꭕ^2^ = 6.6*), the botanical category of seed (ꭕ^2^ = 9.7**), the type of cereals (ꭕ^2^ = 10.5*) and pulses that is produced (ꭕ^2^ = 17.2**). Consequently, in the Sudano-Sahelian zone, the majority of respondents (57.1%) indicated receiving 3 inspections compared to 2 inspections in the highlands zone (53.5%). Also, cereal seeds fields received majorly three inspections (63.8%) compared to two inspections for pulse grains fields (53.5%). These different controls of seed fields by experts help ensure the production of quality seeds. The major challenges faced by seed producers differ between AEZs (ꭕ^2^ = 96.9***), the botanical category of seed (ꭕ^2^ = 30.8***), the cereal types (ꭕ^2^ = 70.1***) and pulses type produced (ꭕ^2^ = 134.3***). Consequently, in the Sudano-Sahelian zone, the major challenge faced by producers is the lack of labour (33.7%); while the lack of funds (31.9%) dominated in the highlands. However, the high cost of inputs is the second major challenge in both AEZs. A difficulty of conserving seeds is a significant challenge in the highlands zone (20.6%) and does not seem to be a major problem in the Sudano-Sahelian zone. Also, the lack of funds is much more significant in the cultivation of pulses seeds compared to cereals grains (Table 4).

**Table 4:** Characteristics of the seed system sustainability in SSZ and WHZ of Cameroon, expressed as a percentage.

Variables	Modalities	Agro-ecological zone			Botanical category			Cereal				Pulse				
		SSZ	WHZ		Cereal	Pulse		Maize	Rice	Sorghum		Peanut	Soybean	Cowpea	Common bean	
		N = 133	N = 43		N = 105	N = 71		N = 60	N = 14	N = 31		N = 13	N = 14	N = 32	N = 12	
				Chi-square			Chi-square				Chi-square					Chi-square
Distance between production and storage site	1-5 km	51.88	44.19	3.4^NS^	40.95	63.38	9.5^*^	38.33	28.57	51.61	17.9^**^	84.62	71.43	46.88	75.00	10.3^NS^
	6-10 km	19.55	25.58		25.71	14.08		18.33	64.29	22.58		0.00	7.14	21.88	16.67	
	11-15 km	13.53	6.98		15.24	7.04		16.67	7.14	16.13		0.00	7.14	9.38	8.33	
	> 15 km	15.04	23.26		18.10	15.49		26.67	0.00	9.68		15.38	14.29	21.88	0.00	
Quantity of seed per hectare	≤ 20	65.41	44.19	49.3^***^	78.10	33.80	39.8^***^	88.33	0.00	93.55	105.7^***^	15.38	21.43	59.38	0.00	59.5^***^
	]20;30]	8.27	13.95		8.57	11.27		11.67	0.00	6.45		7.69	35.71	6.25	0.00	
	]30;50]	22.56	0.00		9.52	28.17		0.00	71.43	0.00		38.46	35.71	31.25	0.00	
	> 50	3.76	41.86		3.81	26.76		0.00	28.57	0.00		38.46	7.14	3.13	100.00	
Yield	≤ 1	23.31	6.98	39.3^***^	1.90	45.07	53.9^***^	0.00	0.00	6.45	34.0^***^	46.15	28.57	59.38	25.00	29.5^**^
	]1;2]	54.89	41.86		59.05	40.85		53.33	71.43	64.52		53.85	50.00	31.25	41.67	
	]2;3]	18.80	23.26		27.62	8.45		33.33	0.00	29.03		0.00	21.43	9.38	0.00	
	]3;4]	0.75	27.91		8.57	5.63		13.33	7.14	0.00		0.00	0.00	0.00	33.33	
	> 4	2.26	0.00		2.86	0.00		0.00	21.43	0.00		0.00	0.00	0.00	0.00	
Selling price (F CFA)	[300;600]	27.07	27.91	2.0^NS^	43.81	2.82	107.9^***^	43.33	14.29	58.06	90.4^***^	7.69	7.14	0.00	0.00	17.5^*^
	]600;900]	29.32	30.23		44.76	7.04		56.67	0.00	41.94		0.00	14.29	9.38	0.00	
	]900;1200]	39.10	41.86		11.43	81.69		0.00	85.71	0.00		61.54	78.57	84.38	100.00	
	> 1200	4.51	0.00		0.00	8.45		0.00	0.00	0.00		30.77	0.00	6.25	0.00	
All seed sold	Yes	40.60	88.37	29.7^***^	53.33	50.70	0.1^NS^	60.00	78.57	29.03	12.0^**^	38.46	50.00	40.63	91.67	10.1^*^
	No	59.40	11.63		46.67	49.30		40.00	21.43	70.97		61.54	50.00	59.38	8.33	
Future of unmarked seeds	No leftovers	54.89	88.37	15.6^***^	64.76	60.56	0.3^NS^	66.67	100.00	45.16	12.9^**^	53.85	57.14	53.13	91.67	5.9^NS^
	Human consumption	45.11	11.63		35.24	39.44		33.33	0.00	54.84		46.15	42.86	46.88	8.33	
Number of inspections per campaign	2	35.34	53.49	6.6^*^	30.48	53.52	9.7^**^	33.33	0.00	38.71	10.5^*^	53.85	50.00	37.50	100.00	17.2^**^
	3	57.14	46.51		63.81	40.85		61.67	100.00	51.61		46.15	50.00	50.00	0.00	
	4	7.52	0.00		5.71	5.63		5.00	0.00	9.68		0.00	0.00	12.50	0.00	
Major challenges	Bird attacks	2.33	0.00	96.9^***^	0.92	0.00	30.8^***^	0.00	7.41	0.00	70.1^***^	0.00	0.00	0.00	0.00	134.3^***^
	Inputs high cost	23.26	28.79		29.49	23.81		27.56	40.74	28.57		25.00	26.92	26.98	5.88	
	Conservation	0.00	20.62		13.36	19.05		11.02	0.00	23.81		20.00	23.08	22.22	0.00	
	Administration	13.95	0.00		0.00	9.52		0.00	0.00	0.00		10.00	3.85	1.59	47.06	
	Selling	0.00	1.95		1.84	0.79		1.57	0.00	3.17		5.00	0.00	0.00	0.00	
	Mother seeds unavailable	4.65	1.17		2.30	1.59		3.15	0.00	1.59		0.00	0.00	1.59	5.88	
	Lack of funds	16.28	31.91		26.27	30.95		27.56	25.93	23.81		30.00	42.31	31.75	11.76	
	Lack of labour	33.72	15.56		23.96	13.49		25.98	25.93	19.05		10.00	3.85	15.87	23.53	
	Lack of land	5.81	0.00		1.84	0.79		3.15	0.00	0.00		0.00	0.00	0.00	5.88	

***: Significant at 0.001 probability level, **: Significant at 0.01 probability level, *: Significant at 0.05 probability level, ^NS^: Not significant.

**Table 5:** Variety and species diversity in the seed production system in SSZ and WHZ of Cameroon.

Modalities			Agro-ecological zone		
Variety	Species	General	SSZ	WHZ	
		N = 176	N = 133	N = 43	
CMS 9015	Maize	10.23	13.53	–	
CMS 8501	Maize	3.41	4.51	–	
TZEE	Maize	3.41	4.51	–	
CMS 8704	Maize	1.70	2.26	–	
CMS 2019	Maize	0.57	0.75	–	
CMS 8806	Maize	0.57	0.75	–	
SHABA	Maize	0.57	–	2.33	
CHC 201	Maize	9.66	–	39.53	
CHC 203	Maize	2.84	–	11.63	
CHC 202	Maize	1.14	–	4.65	
NERICA L56	Rice	0.57	0.75	–	
NERICA 8	Rice	1.14	0.75	2.33	
NERICA 3	Rice	4.55	5.26	2.33	
NERICA L36	Rice	1.70	2.26	–	
IR15-MA02	Rice	1.70	2.26	–	
IR15-MA033	Rice	1.70	2.26	–	
S35	Sorghum	8.52	11.28	–	
ZOUAYE	Sorghum	5.68	7.52	–	
CS 54	Sorghum	2.27	3.01	–	
DAMOUGARI	Sorghum	0.57	0.75	–	
CUILLON	Sorghum	0.57	0.75	–	
LORI	Cowpeas	9.09	12.03	–	
FEKEM	Cowpeas	5.11	6.77	–	
MTA 32	Cowpeas	0.57	–	2.33	
METCHICHA	Peanut	0.57	–	2.33	
JL 24	Peanut	4.55	5.26	2.33	
MANIPENTA	Peanut	1.70	2.26	–	
ICGV	Peanut	0.57	0.75	–	
TGX 1835-10E	Soybean	0.57	–	2.33	
TGX 1910-14F	Soybean	3.41	4.51	–	
HOULA	Soybean	2.27	3.01	–	
TGX 1448-2E	Soybean	1.70	2.26	–	
MAC 32	Common bean	0.57	–	2.33	
GLP 190	Common bean	1.70	–	6.98	
DOR 701	Common bean	1.14	–	4.65	
MEX 142	Common bean	0.57	–	2.33	
NUV 109−2	Common bean	0.57	–	2.33	
PNN	Common bean	0.57	–	2.33	
TY	Common bean	0.57	–	2.33	
FEP 192	Common bean	0.57	–	2.33	
					Hutcheson t-test
H’ Variety		3.22	2.86	2.31	2.57*
H’ Specie		1.76	1.68	1.13	3.59***
Variety richness		40.00	25.00	19.00	
Specie richness		7.00	6.00	6.00	

***: Significant at 0.001 probability level, *: Significant at 0.05 probability level, N: total number.

### Cultivar and species diversity within seed production systems

**Fig 2:**
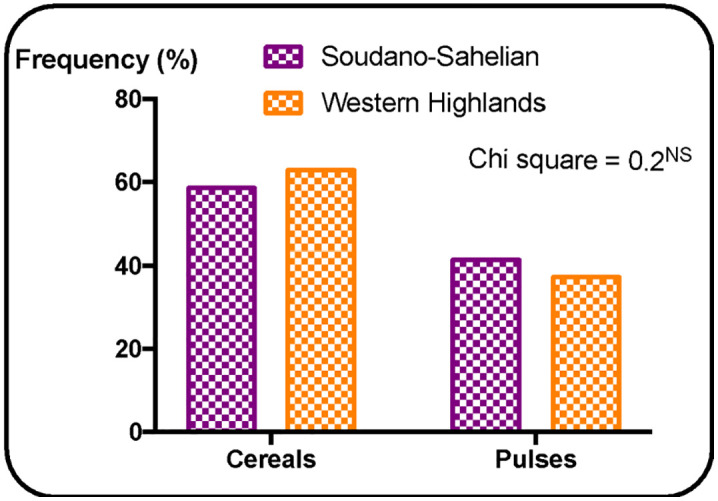
Frequency (%) of appearance of different botanical seed categories according to agroecological zones.

**Fig 3:**
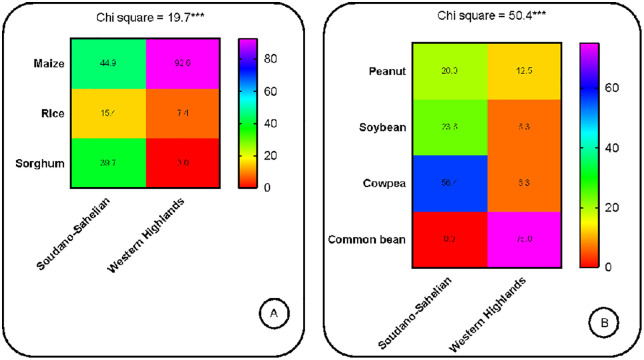
Colour mapping distribution of cereals (A) and pulses (B) according to agro-ecological zones (percentage of respondents).

**Fig 4:**
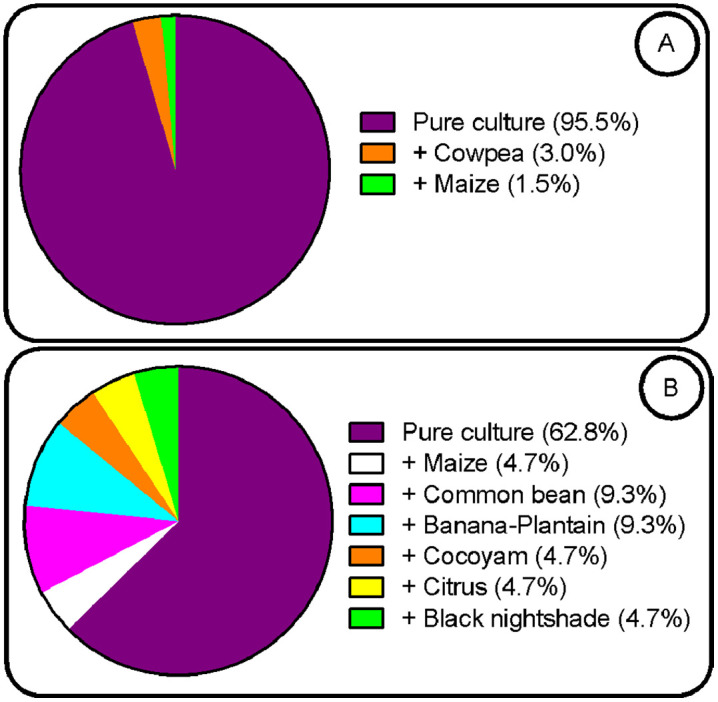
Percentage of of respondents for adoption of cropping system and crops association in seed production systems in Sudano-Sahelian (A) and western highlands (B) zones of Cameroon.

Regarding diversity, in terms of species, the Sudano-Sahelian zone is more diverse (maize, rice, sorghum, cowpea, peanut and soybean) with a Shannon index of 1.68 compared to the highlands zone (maize, rice, cowpea, peanut, soybean and bean) with a Shannon index of 1.13 (t = 3.59***, Table 5). Both zones have the same specific richness of 6. At the varietal level, the Sudano-Sahelian zone remains more diverse with a Shannon index of 2.86 compared to the highlands zone which has a Shannon index of 2.31 (t = 2.57*, Table 5). Also, with 25 varieties used in seed production, the Sudano-Sahelian zone is richer than the highlands zone which has 19. Almost all varieties used in seed production in the Sahelian zone are not used in the Highlands zone and reciprocally. Only NERICA 8, NERICA 3 and JL 24 seeds are simultaneously produced in both agroecological zone. The CMS 9015 variety is the most produced maize variety in the Sudano-Sahelian zone (13.5%) while in the highlands zone it is rather the CHC 201 variety (39.5%). There are no common maize varieties produced simultaneously in the two AEZs. Sorghum which is a crop specific to the Sudano-Sahelian zone has multiplication proportions of 23.4%, close to that of maize in the zone (26.4%). Variety named S35 is the most multiplied sorghum seed (11%). Two cowpea varieties are produced in the Sudano-Sahelian zone and representing 18.8% of seed production and are LORI (12.0%) and FEKEM (6.8%). Only one variety (MTA 22) representing 2.3% of seed production is produced in the highlands zone. Common beans seed production activities were only found in the highlands zone with GLP 190 variety being the most multiplied and accounting for 7.0% of the total seed production in the site. Cereal seeds are produced more than pulses, this in both AEZs (Fig 2). Maize being the most produced cereal in both zones, it is noted a very high proportion in the highlands zone (92.6%) in comparison with the Sudano-Sahelian zone (44.9%, Fig 3). The most produced in the Sudano-Sahelian zone is cowpea (56.4%) against common bean (75.0%) in the highlands zone (Fig 3). Seed cultivation system depends strongly on the AEZs. Seeds are mainly produced in a pure system in the Sudano-Sahelian zone (95.5%) and in the highlands zone (62.8%). However, a more marked crop association is operated in the highlands zone (Fig 4).

### Correlation between variables of the seed production

**Fig 5:**
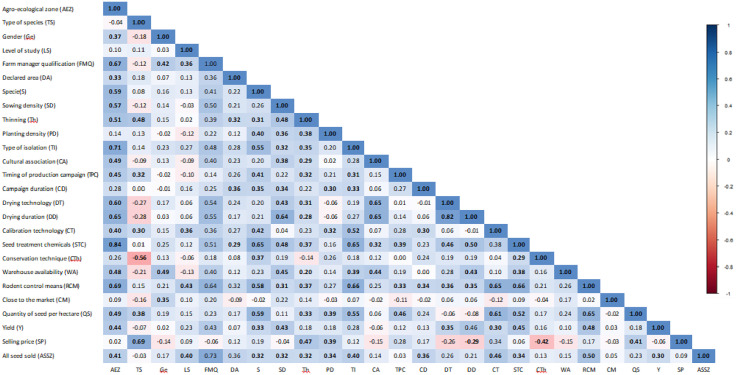
Correlation coefficients between variables of the seed production systems (values in bold are significant at 5% probability level).

The correlation coefficient analysis revealed strong relationships, either positive or negative, among several variables in the seed production system (Fig 5). Among the variables, AEZs correlated significantly (p < 0.05) with the majority of seed production systems, highly with usage of pesticides (r = 0.8), main employment of the producer (r-0.7), type of isolation (r = 0.7) and means of pest control (r = 0.7). Other highly correlations were observed between drying techniques and drying duration (r = 0.8), botanical category of seed and selling price (r = 0.7), main occupation of producer and seed flow (r = 0.7), crop association and drying duration (r = 0.7). Means of pest control correlated positively with the type of isolation (r = 0.7), calibration technology (r = 0.7) and usage of pesticides (r = 0.7). Negative correlations were observed between the botanical category of seed and conservation technique (r = −0.6), drying technology (r = −0.3) and drying duration (r = 0.3). Also, selling price correlates negatively with conservation techniques (r = −0.4), drying technology (r = −0.3) and drying duration (r = −0.3). Other seed production variables showed either positive or negative weak to moderate associations as presented in Fig 5.

## Discussion

Results revealed that seed multiplication activities are dominated by men. This result is similar to the work carried out by Westengen et al. [23]. However, there is a significant proportion of women who practice this activity in the highlands zone compared to the Sudano-Sahelian zone. This gender disproportion between the agroecological zones is certainly due to cultural differences in these two different zones. Indeed, in the Sudano-Sahelian zone, which is predominantly Muslim in culture, tradition would have it that women are the guardians of the home while men take care of finances [29,30]. As a result, women are not called upon to carry out income-generating activities. Regarding the level of education, the majority of seed producers have a higher level of education. This is due to the fact that the activity of multiplying improved seeds is formal and requires specialized skills, found through university training. These results corroborate those of Kirui [31] who reveal this fact in several tropical African countries that include Ethiopia, Malawi, Nigeria and Tanzania. Concerning the categories of seed companies, the surveys revealed that in the Sudano-Sahelian zone, multipliers are more organized in seed production cooperative societies while in the highlands zone, they are much more organized in common initiative groups. The choice of the cooperative society in the Sudano-Sahelian zone makes it possible to reach markets that are very far from the production areas and also offers the possibility to producers to receive news and skills within the cooperative. The choice of initiative groups in the highlands area comes from the fact that the markets are usually close and producers with a high level of education have the skills to mastering these markets. Producers in the Sudano-Sahelian area generally have declared farm areas larger than those of producers in the highlands area. This is because the Sudano-Sahelian area is much larger than the highlands area with lands more available [32].

The major part of mother seeds come from the IRAD stations. It is explained by the fact that the production of foundation seeds necessary for multiplication to obtain certified seeds is a process that requires specialized skills and is generally under the supervision of approved research stations. In addition, the national seed council regulates the activity in order to ensure the production of high-quality seeds and therefore, protecting farmers against counterfeit seeds as highlighted by Guei et al. [33]. Regarding the useful surface area, the survey results show that large useful surfaces of between 0.3 and 0.4 m^2^ per pocket are mainly used in the Sudano-Sahelian zone and in cereal production. In contrary, smaller useful surfaces of between 0 and 0.1 m^2^ are mainly used in the highlands zone and in pulses growing. These results are because of the difference in requirements between seed type and the availability of land. Indeed, cereal cultivation requires a little more space than pulse production [34]. Consequently, the sowing density of pulses is higher than that of cereals. Also, we observe the use of high sowing density in the Sudano-Sahelian zone compared to the highlands zone. However, there is a balance between planting densities in the two agroecological zones. This is due to the practice of thinning in the Sudano-Sahelian zone which thus brings the planting density back into balance with the highlands zone. In order to ensure the purity at the varietal level and even the species level of their seed, producers must practice isolation. Spatial isolation is mainly used in the Sudano-Sahelian zone, while in the highlands zone it is the temporal isolation. This latter practice, widely used in the highlands zone, is absent in the Sudano-Sahelian zone. Indeed, since the growing season is relatively short in the Sudano-Sahelian zone, seed producers cannot afford to practice temporal isolation. Following the failure of some producers to practice spatial isolation due to the unavailability of land, they opt for the sharing seeds with their close neighbours in order to be able to produce seeds of quality during the growing season. Although land is unavailable in the highlands zone, this latter practice is non-existent there. Indeed, the long duration of the growing season offers producers the possibility of shifting sowing dates in order to ensure the isolation of their plot (temporal isolation). Sowing is mainly carried out at the beginning of the campaign in both AEZs. However, in the highlands zone, there is a fairly high proportion of producers who sow after the start of the rains (41.9%), compared to producers in the Sudano-Sahelian zone (11.3%). This is due to the relatively short duration of rain in the Sudano-Sahelian zone compared to the highlands zone. This fact has an impact on the duration of the campaign which translates into an average duration of 5 months in the Sudano-Sahelian zone compared to 4 months in the highlands zone.

Survey results showed in terms of drying technology that on-field drying is the most widely used technology in both zones although its use is more extensive in the Sudano-Sahelian zone. However, we noted the marked use of cribs and granaries in the highlands zone, non-existent in the Sudano-Sahelian zone. The significant choice of on-field drying in the Sudano-Sahelian zone stems from the fact that they only have one agricultural season with short raining duration, 3 months on average. Consequently, the products reach maturity at the beginning of the dry season, giving them the possibility of letting drying directly in the field. Contrary in the highlands zone, the rains extend over 9 months on average offer the possibility of carrying out two agricultural seasons [32]. The first season ends during the rainy season and therefore, there is need of structures such as cribs and granaries with roofs for rain protection in order to dry the product optimally. The second season, which ends at the start of the dry season, gives the possibility of leaving the dried products in the field. Drying time is shorter in the Sudano-Sahelian zone compared to the highlands zone. This is associated to the temperatures, much higher in the former compared to the latter as reported by IRAD (2008). Drying time is longer for cereals compared to pulses. This is due to the humidity amount for the conservation of pulses which is generally higher than that of cereals [35,36]. The results reveal the non-use of chemicals in controlling pests by the majority of producers in the two AEZs. The low use of chemicals is due to the possibility of using non-commercialized seeds in feeding humans or animals. Ambient conservation is the most used technique in both zones because of the low incomes of seed producers and the high cost of materials required for hermetic conservation. Storage warehouses are available for the majority of seed producers in the Sudano-Sahelian zone compared to producers in the highlands zone. Being organized into cooperatives in the Sahelian zone, facilitates to raise funds required to build standard storage warehouse. Seeds are stored there for 5 months on average. Longer storage periods of more than 7 months are observed at the contrary of shorter periods of 3 months observed in the highlands zone. This is associated to the existence of only one agricultural campaign in the Sudano-Sahelian zone which extends the storage duration, compared to two campaigns in the highlands zone which reduces time in conserving seeds.

At the species and the variety level, diversity estimates showed high values with the Sudano-Sahelian zone more diverse than the highlands zone. This marked diversity is important for strengthening the sustainability of agricultural and seed systems because improving resilience, productivity and ecosystem services. Diversified cropping systems can better absorb shocks and improve soil health through nutrient cycling and carbon capture [37,38]. The distance between the seed production site and the market of less than 5 km is strongly represented in both ZAEs. This short distance has the effect of strengthening the sustainability of the systems by reducing losses during transport, promoting accessibility and low cost of seeds. In the highlands, larger quantities of seeds are used compared to the Sudano-Sahelian zone. Similarly, the cultivation of pulses requires a larger quantity of seeds, generally greater than 50 kg/ha, than that of cereals, which is on average 20 kg/ha. In cereals, the cultivation of maize and sorghum requires quantities of seeds between 10 and 20 kg/ha compared to high quantities generally greater than 30 kg/ha for rice. These quantities of seeds used are reasonable and correspond to the quantities used in the seed production systems [39]. These quantities used in accordance with the regulations and literature allow to avoid wasting resources and therefore to strengthen the sustainability of the system. Yields are relatively low in both agroecological zones and are between 1 and 2 t/ha. However, even lower yields, less than 1 t/ha are observed in the Sudano-Sahelian zone while yields greater than 2 t/ha are observed in the highlands zone. While cereals mostly have yields between 1 and 2 t/ha, pulses on the other hand have yields mostly less than 1 t/ha. But beans in particular have a yield greater than 1 t/ha. These average yield values are similar to the average values in sub-Saharan Africa [40,41]. However, these values remain lower than the average yield of cereals in the world which is 5 t/ha except for sorghum which is at 1.6 t/ha [13].

## Conclusion

The characterization of the cereal and pulse seed system shows an activity dominated by men with a higher level of education. The companies are mainly organized in cooperatives with large farm sizes in the Sudano-Sahelian zone, while in the highlands zone, they are mainly organized in CIGs with small farm sizes. The foundation seeds used by seed producers are mainly from IRAD and correspond to a quantity of less than 20 kg/ha for cereals against more than 30 kg/ha for pulses. Thinning, which is specific to the Sudano-Sahelian zone, allows it to bring its planting density back to a balance with the highlands zone. To avoid contamination, spatial isolation is mainly observed in the Sudano-Sahelian zone against temporal isolation in the highlands zone. This leads to sowing after the start of the rains in the highlands, while in the Sudano-Sahelian zone sowing is done as soon as the rains start. The duration of the crop season is therefore longer in the Sudano-Sahelian zone compared to the highlands. Open-field drying is mainly used in both AEZs. However, in the highlands, the use of cribs and granaries in addition to on-field drying makes it possible to extend the drying duration beyond 8 months. Seed calibration is mainly carried out using sieves with different mesh sizes. While seeds stored in the Sudano-Sahelian zone are generally not treated with pesticides, in the highlands zone, on the other hand, there is substantial use of pesticides such as momtaz for cereals and antouka for pulses. Seeds are generally stored ambiently in both AEZs. There is a proximity between production sites and marketplaces, facilitating accessibility. While in the highlands zone, the majority of producers generally sell all of their seeds, this is not the case in the Sudano-Sahelian zone, with unmarketed seeds mainly redirected towards human and animal feeding. The major challenges facing seed producers are the lack of labour and funds. The Sudano-Sahelian zone is more diversified in seed production activities compared to the highlands zone where the practice of crop association in seed production system is pointedly observed.

## Supporting information

S1 File Minimal data set (Fotso Ngangoua et al.). (ꭕLSꭕ)
